# Noradrenaline modulates neuronal and perceptual visual detectability via β-adrenergic receptor

**DOI:** 10.1007/s00213-021-05980-y

**Published:** 2021-09-21

**Authors:** Keisuke Tsunoda, Akinori Y. Sato, Ryo Mizuyama, Satoshi Shimegi

**Affiliations:** 1 Laboratory of Brain Information Science in Sports, Center for Education in Liberal Arts and Sciences, Osaka University, Toyonaka, Osaka Japan; 2 Laboratory of Brain Information Science in Sports, Graduate School of Frontier Biosciences, Osaka University, Toyonaka, Osaka Japan; 3 Present Address: Medical Innovation Center, Graduate School of Medicine, Kyoto University, Kyoto, Japan; 4 Present Address: Laboratory of Cellular Pharmacology, Graduate School of Pharmaceutical Sciences, Nagoya University, Nagoya, Japan

**Keywords:** Noradrenaline, β-adrenergic receptor, Primary visual cortex, Visual detectability, Rat

## Abstract

**Rationale:**

Noradrenaline (NA) is a neuromodulator secreted from noradrenergic neurons in the locus coeruleus to the whole brain depending on the physiological state and behavioral context. It regulates various brain functions including vision via three major adrenergic receptor (AR) subtypes. Previous studies investigating the noradrenergic modulations on vision reported different effects, including improvement and impairment of perceptual visual sensitivity in rodents via β-AR, an AR subtype. Therefore, it remains unknown how NA affects perceptual visual sensitivity via β-AR and what neuronal mechanisms underlie it.

**Objectives:**

The current study investigated the noradrenergic modulation of perceptual and neuronal visual sensitivity via β-AR in the primary visual cortex (V1).

**Methods:**

We performed extracellular multi-point recordings from V1 of rats performing a go/no-go visual detection task under the head-fixed condition. A β-AR blocker, propranolol (10 mM), was topically administered onto the V1 surface, and the drug effect on behavioral and neuronal activities was quantified by comparing pre-and post-drug administration.

**Results:**

The topical administration of propranolol onto the V1 surface significantly improved the task performance. An analysis of the multi-unit activity in V1 showed that propranolol significantly suppressed spontaneous activity and facilitated the visual response of the recording sites in V1. We further calculated the signal-to-noise ratio (SNR), finding that the SNR was significantly improved after propranolol administration.

**Conclusions:**

Pharmacological blockade of β-AR in V1 improves perceptual visual detectability by modifying the SNR of neuronal activity.

## Introduction

Noradrenergic neurons in the locus coeruleus (LC) are part of the reticular ascending system (Dahlstroem and Fuxe 1964), secrete noradrenaline (NA) to the whole brain, elevate arousal level, and regulate various brain functions such as memory, stress, and sensation including vision (Waterhouse and Navarra 2019).

NA is known to activate three adrenergic receptor (AR) subtypes (*α*_1_, *α*_2_, and β) and differently modulates perception or cognition via each (Ramos and Arnsten 2007; Sara 2009). In vision, some studies reported that β-AR modulates perceptual visual sensitivity, but the effects were inconsistent. Treviño et al. (2019) reported that the local administration of β-AR agonist on the primary visual cortex (V1) worsens the task performance of mice performing the two-alternative forced-choice (2-AFC) visual discrimination task in a water maze. On the other hand, Mizuyama et al. (2016) showed that the systemic administration of a β-AR antagonist impairs the visual detectability of rats performing the 2-AFC visual detection (VD) task in an operant chamber. In other words, both the activation and deactivation of β-AR have been reported to impair visual sensitivity. Thus, it remains unclear how the activation of β-AR modulates perceptual visual sensitivity.

Several electrophysiological studies showed that NA variously modulates the neuronal activity or signal-to-noise ratio (SNR) in sensory areas via different AR subtypes (Sato et al. 1989; Devilbiss and Waterhouse 2000; Atzori et al. 2016; Jacob and Nienborg 2018). Sato and colleagues performed extracellular recordings in V1 of anesthetized cats and tested the iontophoretic administration of α_1_-, α_2_-, and β-AR antagonists to study the functional roles of these AR subtypes in V1. They found that the activation of α_1_- and α_2_-ARs mainly facilitated both the visual response and spontaneous firing, but the activation of β-AR facilitated or inhibited the visual response of each cell. These neuromodulatory effects in V1 could contribute to the improvement or impairment of perceptual sensitivity in behaving animals (Mizuyama et al. 2016; Treviño et al. 2019). However, it still remains unknown how NA modulates the neuronal activity in V1 via β-ARs or how the adrenergic modulation affects the visual perception of awake animals.

To investigate the above-mentioned points, we simultaneously assessed perceptual visual detectability and the multi-unit activity (MUA) of V1 in rats performing a VD task and examined the effects of the local administration of a β-AR antagonist, propranolol, in V1.

## Materials and methods

### Animals and surgery

All experimental protocols were approved by the Research Ethics Committee of Osaka University (Permit Number: 28–074-000) and carried out in compliance with the policies and regulations of the guidelines approved by the Animal Care Committee of the Osaka University Medical School and National Institutes of Health guidelines for the care of experimental animals. Male Long-Evans rats (250–350 g; *n* = 5; Japan SLC Inc., Shizuoka, Japan) were used and kept on a reversed light–dark cycle (lights off 9:00; lights on 21:00) under controlled temperature (22–24 °C). We performed all behavioral training and experiments between 9:00 and 21:00. The surgical protocol of the head-plate implantation to rats was performed based on previous studies (Kimura et al. 2012; Soma et al. 2017, 2019). First, we anesthetized rats with isoflurane (5% for induction and 2–2.5% for maintenance) using an anesthesia apparatus (KN-1071, Natsume Seisakusho) and maintained the body temperature of the animals during the operation at 37 °C using an animal warming device (TP-500, Gaymer) on a stereotaxic frame (SR-10R-HT, Narishige). A stainless steel head plate (CFR-2, Narishige) was attached on the skull by a stainless steel self-tapping screw (M1.2 × 3 mm), acting as an anchor, and dental resin cement (Super-Bond C&B, Sun Medical; Unifast II, GC Corporation). The reference and ground electrodes (Teflon-coated silver wire, A-M Systems, Φ125 μm) were implanted above the cerebellum. For 1 week after the surgery (recovery period), the antibiotic enrofloxacin (Baytril 10% oral solution, Bayer) was mixed with drinking water at 400 mg/L to prevent infection.

After the recovery period, drinking water was restricted. The restriction was maintained through the experiment period, during which rats were allowed to drink water by performing the VD task, and food was freely available in the cage (Tsunoda et al. 2019; Sato et al. 2020; Soma et al. 2021). Rats were given water as needed to maintain at least 85% body weight at the start of the task training.

### Behavioral task

**Fig. 1:**
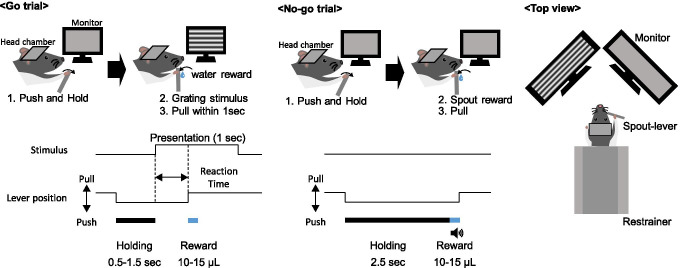
Schematic diagram of the VD task. The rats voluntarily started each trial by pushing the spout-lever in both go and no-go trials. In go trials, the rats had to push and hold the spout-lever with the right forelimb for a random holding period (0.5 ~ 1.5 s), and then visual stimuli were presented for 1 s. The rats had to pull the spout-lever within the presentation period to receive the water reward. In no-go trials, the rats had to push and hold the spout-lever for 2.5 s, and then the hit sound and water reward were given. Monitors were placed on the left and right sides of the rats, but the visual stimulus presentation side was fixed for a daily session

We developed a go/no-go VD task to measure the perceptual visual detectability and neuronal activity of rats simultaneously (Fig. 1). Rats were head-fixed using a stereotaxic apparatus (SR-10R-HT, Narishige), and a spout-lever (OPR-SPL-RM, Ohara Medical Industry, Tokyo, Japan) that integrates a reward spout and manipulation lever was placed in front of their mouths. Gamma-corrected LCD monitors (ProLite G2773HS-2, Iiyama, 144 Hz, mean luminance: 30 cd/m^2^) were placed on the left and right front side of their heads (Fig. 1). Control of the VD task, including generation of the visual stimuli, the judgment of task success/failure, and volume control of the reward (water) dispensation, was performed with custom software written in MATLAB (MathWorks) and Psychtoolbox (Brainard 1997).

#### Go/no-go visual detection task

In the VD task, the rats had to manipulate the spout-lever during the visual stimulus presentation period to obtain the water reward. The task consisted of “go” trials (visual stimulus presentation) and “no-go” trials as a catch trial (no visual stimulus presentation), which allowed us to confirm that the rats were correctly performing the task.

In the go trials, the rats spontaneously started each trial by pushing the spout-lever with the right forelimb and holding it for 0.5–1.5 s (holding period) until the full-screen drift grating stimulus (visual angle, 90 × 65°; spatial frequency, 0.1 cycles/degree (cpd); temporal frequency, 2 Hz; grating orientation, horizontal; drift direction, downward) was presented on one of two monitors for 1 s based on the recording hemisphere. For example, the stimulus was presented on the left monitor, when we recorded from the right V1. The holding period was randomly chosen on a uniform distribution for each trial. The rats had to pull the spout-lever to get the water reward within the stimulus presentation period. If they performed correctly (“hit”), the water reward (10–15 μL) and a hit sound (5000 Hz, 0.15 s) were given. If they did not pull the lever within the stimulus presentation period, the trial was judged as a “Miss” trial, in which a miss sound (4000 Hz, 0.15 s) and a small amount of water reward (3–7 μL, 30–50% of the hit reward) were dispensed to maintain the rats’ motivation. After a random inter-trial interval (ITI) ranging from 0.2 to 0.8 s, the rats could start the next trial by pushing the spout-lever. If they pulled the lever before the stimulus presentation, the trial was judged as a “false alarm” trial, leading to a false alarm sound (2500 Hz, 1 s) and lengthening of the ITI to 3–5 s.

In no-go trials, the rats spontaneously started each trial by pushing the spout-lever with the right forelimb and holding it for 2.5 s. Then, the water reward (10–15 μL) and hit (5000 Hz, 0.15 s) sound were presented. If the rats pulled the lever before the reward dispensation, the trial was judged as a “false alarm” trial, leading to the same false alarm sound (2500 Hz, 1 s) and ITI lengthening as above.

#### Perceptual contrast detectability measurement

Grating contrast detectability was measured in the VD task, and several levels of stimulus contrast were tested. The VD task was block-designed, with one block consisting of 10 trials containing 40% go trials and 60% no-go trials. The type of trial (go/no-go) was randomly set without the instruction cue, and the rats were not informed of the trial type at the start of each trial. The rats were taught to push the lever until they detected a visual stimulus (go trials) or water dispensed from the spout-lever (hit in no-go trials or miss in go trials). By adopting this method, we intended to prevent the rats from conducting inappropriate lever manipulation, such as not attending to the visual stimuli or pulling the lever regardless of the stimulus presentation. The grating contrast of each block was constant and changed randomly from block to block. The rats performed 103 ± 14 blocks in 80 min a day (mean ± SD).

In the main experiment, four to six contrast values were tested. Since the perceptual contrast detectability differed for each rat, a relationship between the stimulus contrast and task performance was examined in the preliminary experiment. The contrast values used in the main experiment were selected at or around the chance level (50%) of the task performance for each rat, which enabled us to examine the drug’s positive and negative modulatory effects.

#### Behavioral task training

The rats were trained 0.5–2 h per day and typically learned the VD task within 2 weeks. On the first day of training, for the purpose of acclimatization to a stereotaxic apparatus, the rats under the head-fixed condition received water by licking the spout-lever near their mouth. The training lasted about 30–60 min until the rats stopped licking voluntarily. The rats were able to easily acclimatize to the head-fixed condition by performing this licking task.

From the 2nd to 4th days, the rats learned the association between a visual stimulus and to pull the spout-lever to obtain a water reward. The rats spontaneously pushed the spout-lever with the right forelimb, which was the trigger of the visual stimulus presentation, and a full-screen drift grating stimulus appeared on the monitor at the right side (visual angle, 90 × 65°; spatial frequency, 0.001 cpd; temporal frequency, 5 Hz; drift direction, downward; contrast, 100%). When the rats pulled the spout-lever during the stimulus presentation, the reward was dispensed from the tip of the spout-lever. In the early phase of the learning period, the visual stimulus was presented just after the rats pushed the spout-lever. The time interval between the push and the stimulus presentation was gradually increased from 0.01 to 1.5 s to make the rats learn to keep pushing the lever (holding). When the rats pulled the spout-lever before the stimulus appeared on the display, no reward was given. Thereby, the rats learned the association between the stimulus presentation and lever manipulation. The rats performed this training task for 3 days, and all of them learned to keep holding the spout-lever for more than 1.5 s until the visual stimulus presentation.

On the 5th and 6th days, the rats learned to pull the spout-lever immediately after they detected the stimulus. The visual stimulus conditions were changed from the grating stimulus with low spatial and temporal frequency, which resembled a blinking light, to approximate the measurement condition of the contrast sensitivity as much as possible (visual angle, 90 × 65°; spatial frequency, 0.1 cpd; temporal frequency, 2 Hz; drift direction, leftward; contrast, 100%). The time interval between the lever-pushing and the subsequent stimulus presentation was randomly changed from 0.5 to 1.5 s, and the stimulus presentation period was fixed to 1 s. Using this training task condition, we prevented the rats from learning the wrong strategy, i.e., the rats just push the lever for a certain trained period (e.g., 2 s) and pull it regardless of the stimulus presentation. In the task, if the rats pulled the lever before or after the stimulus presentation, the rats were punished with an additional 2.1–2.9 s ITI and were not rewarded with water.

From the 7th day of training, no-go trials were included in the training session. Go and no-go trials were shuffled in a daily session, and a session in one day consisted of 30% no-go trials. If the rats pulled the lever before the reward was given in the no-go trials, an additional 3–5 s ITI was given. We judged the rats’ complete learning of the training version of the VD task when the % Hit of go/no-go trials in a session was above 80% for the left and right visual stimulus sides each for 3 successive days. The measurement of perceptual contrast detectability was started after the confirmation of complete learning.

#### Electrophysiological recordings of multi-unit activity (MUA)

After the rats completed the task learning, we performed a second surgery in which tiny holes (1.5–2.0 mm in diameter) were made in the skull and dura mater above V1 (3.7 mm posterior to the bregma, 7.3 mm lateral). We recorded MUA with a 2-shank, 32-channel multi-point silicon electrode (Isomura32-a32, NeuroNexus Technologies; 16 active channels separated 150 μm in each shank and were located from the tip to 1050 μm; the length of the electrode, 7 mm), while the rats were performing the VD task. Electrodes were precisely inserted in V1 using an electric manipulator (SM-21, Narishige). Signals were amplified, filtered, and recorded with a recording system (OmniPlex, Plexon; final gain, 1000; bandpass filter, 0.7 to 8 kHz; sampling rate, 40 kHz) through a 32-channel preamplifier (HST-32 V-G20-GR, Plexon; gain: 20). All event triggers during the VD task (e.g., stimulus onset, spout-lever position) were recorded through the same recording system. All recordings were performed in V1 contralateral to the stimulus presentation side.

#### Spike sorting to denoise MUA

To denoise MUA, spike activity was isolated for each channel using the automatic spike-sorting software KlustaKwik (Rossant et al. 2016). First, a 0.3–8-kHz bandpass filter was applied to the potential waveform data, and a potential change exceeding two standard deviations (SD) from the baseline was detected as a spike. Noise clusters were manually removed using KlustaViewa, and the other clusters in a recording site were combined and defined as a MUA.

### Drug administration

The drug solution was topically administered onto the V1 surface around the electrode recording site (Goard and Dan 2009; Soma et al. 2013). A β-AR antagonist (propranolol, 10 mM, Tokyo Kasei) was dissolved in phosphate-buffered saline (PBS) at pH 7.4, which is euhydric (physiological pH). The drug concentration was determined based on previous studies (Goard and Dan 2009; Soma et al. 2013; Manella et al. 2017) and our preliminary experiments (data not shown) which confirmed if the drug concentration did not affect the execution of the task (e.g., task cessation due to sedation). We tested only one drug concentration (10 mM), because the activation of β-AR causes a monotonical decrease in neuronal activity (Devilbiss and Waterhouse 2000), implying that the β-AR agonist/antagonist causes no concentration-dependent change in the direction of the modulatory effects in the cortex.

First, a microwell was made by gluing a plastic ring to the skull area surrounding the craniotomy. After inserting the electrode in V1, the microwell was filled with PBS. The first 40 min of the VD task with the MUA recording was conducted under PBS condition. Then, PBS was aspirated, and propranolol or PBS (control condition) was administered onto the V1 surface. Subsequently, the VD task was continued for 40 min. We excluded data for 10 trials (0.75 ± 0.09 min) immediately before and after the drug administration from the behavioral and neuronal analysis.

### Experimental designs

The experiments were conducted to investigate the noradrenergic modulation of vision using a single-blind within-subject cross-over design, and the order of drug conditions was randomized and counter-balanced. After the behavioral training was completed, we performed two recording sessions from the left and right V1 of one rat, respectively. In total, 10 pairs (PBS/propranolol) of the recordings from 5 rats were performed. One pair of the recording sessions was excluded from the analysis because V1 was severely damaged. The neuronal and behavioral analyses were performed with the remaining 9 pairs.

### Analysis of data

#### Analysis of behavioral performance

The VD task performance was evaluated as the hit rate and the reaction time in the go trials. The pharmacological effects of propranolol on the performance were quantified by calculating the performance change index (PCI) as below:

$$\mathrm{PCI}=1+\frac{\left(\mathrm{Post}-\mathrm{drug performance}\right)-(\mathrm{Pre}-\mathrm{drug performance})}{\left(\mathrm{Post}-\mathrm{drug performance}\right)+(\mathrm{Pre}-\mathrm{drug performance})}$$

The PCI is a measure that reflects the amount of change before and after drug administration and can prevent factors such as individual differences and inter-day fluctuations from contaminating the drug efficacy. It has been validated as a measure of the rate of change (e.g., percent change of behavioral performances) in human and animal studies that tested noradrenergic drug effects in a sequential experimental design (Navarra et al. 2017; Gelbard-Sagiv et al. 2018) or in animal study that quantified behavioral discriminability (Jun et al. 2020). The drug effect was investigated by comparing the PCI calculated for PBS and for propranolol using the Wilcoxon signed-rank test.

In addition, we analyzed the behavioral data using a two-way analysis of variance (ANOVA) for repeated measures. The factors were drug (saline, propranolol) and conditions (pre, post). When the ANOVA showed a significant interaction between the drug and conditions, a post hoc analysis using the Tukey–Kramer test was performed.

#### Analysis of neuronal activity

To quantify the effects of propranolol on spontaneous activity and visual response separately, the average spike rate within 300 ms windows before (− 300 to 0 ms) and after (0 to + 300 ms) the onset of the visual stimulus was calculated as the spontaneous activity and the visual response, respectively. First, to determine whether the recording sites show significant visual responses, we compared the spontaneous activity and visual response of each recording site by using Wilcoxon rank-sum test. We defined a significantly responded recording site (*p* < 0.05) as a visually responsive recording site (VR-site). Then, the statistical significance of the propranolol modulatory effects was analyzed by comparing the average spike rates before and after the drug administration using the Wilcoxon rank-sum test. Significantly modulated recording sites (*p* < 0.05) were categorized as facilitated or suppressed, and the remaining non-significant sites were categorized as no-effect.

To investigate the effect of propranolol on the goodness of the visual representation in V1, the SNR was calculated using the following formula (Kolta and Reader 1989):

$$SNR=(Rstim-Rspont)/Rspont$$

*R*_stim_ indicates the average firing rate from 0 to 300 ms in the visual stimulus presentation period, and *R*_spont_ indicates the spontaneous firing rate from − 300 to 0 ms before the onset of the visual stimulus. Peri-stimulus time histograms (PSTH; bin width: 1 ms) were temporarily filtered with a Gaussian function of an SD of 50 ms for each condition.

##### Histological observations

After completion of the electrophysiological recording experiments, rats were deeply anesthetized by the intraperitoneal administration of ethyl carbamate (2–3 g/kg, Nacalai Tesque, Japan). After the pain reaction disappeared, the chest was opened, and the heart was perfused with 200 mL of PBS and then with 200 mL of a fixative (4% paraformaldehyde and 0.1 M PBS). After decerebration, the brains were stored in a fixative (4% paraformaldehyde, 0.1 M PBS, 30% sucrose). Using a microtome (REM-700, Daiwa Koiki Kogyo Co., Ltd.), the brains were cut along the sagittal plane of the cerebrum, and a section of thickness 60 μm was prepared. The sections were subjected to nuclear staining using DAPI (4′, 6-diamidino-2-phenylindole, 0.1 μg/mL, Sigma-Aldrich). The electrode was coated with DiI (1,1′-Dioctadecyl-3,3,3′,3′-tetramethylindocarbocyanine, Perchlorate PromoCell, Heidelberg, Germany) before its insertion into the brain. Electrode tracks were then reconstructed and verified with a fluorescence microscope (Eclipse 80i, Nikon).

## Results

To investigate how NA modulates neuronal activity in V1 of awake rats and affects the behavioral visual detectability via β-AR, we conducted a multi-unit recording from V1 of rats performing a VD task and examined the effects of a topically administered β-AR antagonist, propranolol, onto the V1 surface. We quantified the drug effect on behavioral and neuronal activities by comparing pre-and post-drug administration.

Regarding the relationship between stimulus contrast and rat behavioral performance, when the stimulus contrast exceeded 20%, the average task performance (accuracy) of all animals reached 80%. Since Wilder’s law of initial value states that the effect size of the intervention was affected by the initial value (Wilder 1962), we analyzed the data obtained at stimulus contrasts of 20% or less (low contrast condition) and of higher than 20% (high contrast condition) separately.

The behavioral performance of the VD task (accuracy) was first analyzed using a two-way ANOVA for repeated measures. In the low contrast condition, a significant effect was found for the interaction between the drug (saline, propranolol) and time (pre, post), but not for each factor (time, *F*_1, 8_ = 1.58, *p* = 0.24; drug, *F*_1, 8_ = 0.14, *p* = 0.72; interaction, *F*_1, 8_ = 9.49, *p* < 0.05). On the other hand, no significant difference was observed in the effect of each factor or the interaction between factors in the high contrast condition (time, *F*_1, 8_ = 4.63, *p* = 0.06; drug, *F*_1, 8_ = 0.07, *p* = 0.80; interaction, *F*_1, 8_ = 0.03, *p* = 0.86). A post hoc analysis using the Tukey–Kramer test showed no significant difference between before and after drug administration in the low contrast condition (propranolol, *p* = 0.23; saline, *p* = 0.06).

**Fig. 2:**
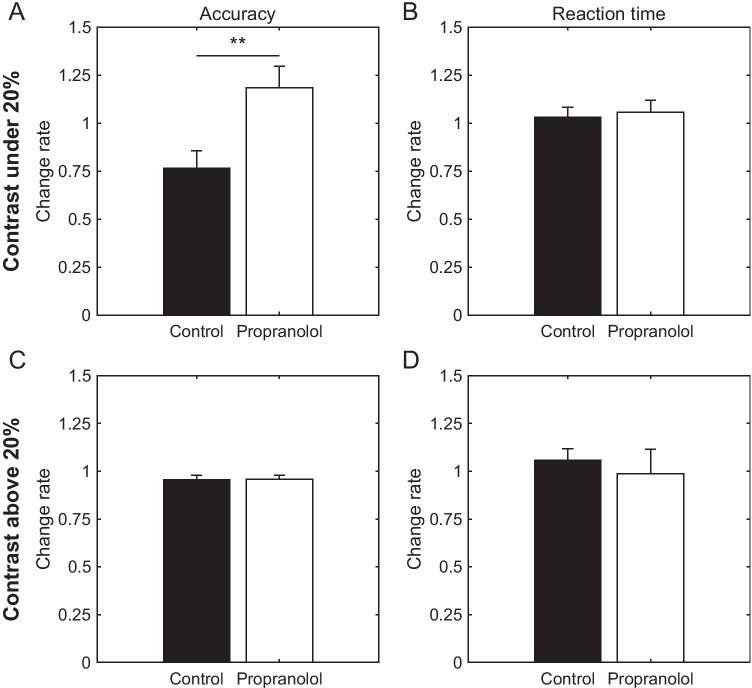
Propranolol effects on behavioral performance of the VD task. Summary of behavioral performance change rates for 9 pairs of experiments on 5 rats. The performance change index (PCI) was calculated as 1 + ((post-drug performance) – (pre-drug performance))/((post-drug performance) + (pre-drug performance)). **A** We calculated the PCI of the hit rates (accuracy) during the pre- and post-drug administration. Propranolol significantly improved the PCI compared to saline with a stimulus contrast of 20% or less (saline, 0.76; propranolol, 1.18, *n* = 9, *Z* =  − 2.55, **p < 0.01, Wilcoxon signed-rank test). The accuracies were calculated as follows: (number (no.) of hit go trials)/(no. of total go trials). **B** Reaction time in go trials. Propranolol had no effect on the PCI of the reaction time with a stimulus contrast of 20% or less (saline, 1.03; propranolol, 1.06, *n* = 9, *Z* = 0.06, *p* > 0.99, Wilcoxon signed-rank test). **C, D** Same as **A** and **B**, but the PCI was calculated using the data with a stimulus contrast above 20%. No significant differences were observed between the saline and propranolol conditions for accuracy (saline, 0.96; propranolol, 0.96, *n* = 9, *Z* =  − 0.41, *p* = 0.68, Wilcoxon signed-rank test) or reaction time (saline, 1.06; propranolol, 0.99, *n* = 9, *Z* = 0.77, *p* = 0.44, Wilcoxon signed-rank test)

However, a significant interaction effect between the drug and time was observed in the low contrast condition. Therefore, the intervention effect was evaluated using PCI (see the “13” section). Figures 2A and C show PCI calculated for accuracy under low and high contrast conditions, respectively. The PCI values were significantly higher with propranolol administration than with saline in the low contrast condition (saline, 0.76; propranolol, 1.18, *n* = 9, *Z* =  − 2.55, **p < 0.01, Wilcoxon signed-rank test, Fig. 2A), but not in the high contrast condition (saline, 0.96; propranolol, 0.96, *n* = 9, *Z* =  − 0.41, *p* = 0.68, Wilcoxon signed-rank test, Fig. 2C). These results show the functional role of β-AR in V1 in the perceptual detectability of low contrast stimuli.

Since the poorer performance following saline administration in the low contrast condition may result from the animal’s fatigue or decline of motivation for the reward due to the sustained concentration on the task, we also analyzed the reaction time (the time between the stimulus onset and pulling the lever). These PCI values were almost 1 for the saline condition, and no significant difference was observed between the saline and propranolol administration (saline, 1.03; propranolol, 1.06, *n* = 9, *Z* = 0.06, *p* > 0.99, Wilcoxon signed-rank test, Fig. 2B), suggesting that the poorer performance following saline administration was not due to fatigue or reduced motivation. Supporting this conclusion was the fact that the PCI values of the reaction time were also around 1 under the high contrast condition (saline, 1.06; propranolol, 0.99, *n* = 9, *Z* = 0.77, *p* = 0.44, Wilcoxon signed-rank test, Fig. 2D).

**Fig. 3:**
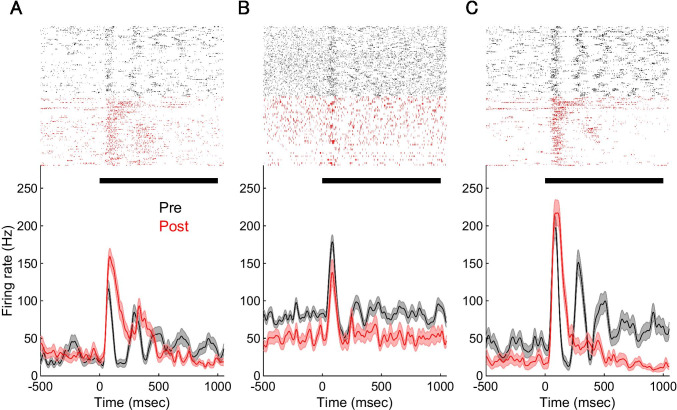
Typical raster plots and PSTHs of three VR-sites in V1. Top, raster plots showing spikes for consecutive trials with pre- (black) and post- (red) propranolol administration. Bottom, PSTHs of pre- and post-propranolol administration. Shaded regions represent SEM. Black horizontal lines above the PSTHs indicate the 1 s visual stimulus presentation period. **A** This MUA showed an enhancement of visually evoked responses and no effect on spontaneous activity. **B** This MUA showed a decrease in spontaneous activity. **C** This MUA showed a decrease in spontaneous activity and an increase in visually evoked responses

Next, to clarify how propranolol administered on V1 improved perceptual visual detectability, we recorded MUA from a total of 125 V1 recording sites in 5 rats and analyzed the neuronal activities, with focus on the low contrast condition based on the findings shown in Fig. 2. We found that 35 and 46 recording sites showed a significant visual response in the saline and propranolol conditions, respectively (see the “14” section). We called these sites visually responsive recording sites (VR-sites). Figure 3 depicts typical examples of raster plots and PSTHs of pre- and post-propranolol administration. After propranolol administration, we observed mainly two types of modulatory effects, the facilitation of visual responses (Fig. 3A, C) and the suppression of spontaneous activities (Fig. 3B, C). The categorization of the modulatory effects was statistically performed using a nonparametric analysis (see the “14” section). Among the 46 VR-sites in the propranolol condition, 11 (24%), 2 (4%), and 33 (72%) were categorized as facilitated, suppressed, and no-effect for visual responses, and 7 (15%), 29 (63%), and 10 (22%) were categorized as facilitated, suppressed, and no-effect for spontaneous activities, respectively. After saline administration, among the observed 35 VR-sites, 1 (3%), 10 (28%), and 24 (69%) were categorized as facilitated, suppressed, and no-effect for visual responses, and 15 (43%), 5 (14%), and 15 (43%) were categorized as facilitated, suppressed, and no-effect on spontaneous activities, respectively.

It is possible that the drug effects followed a concentration gradient, causing differential modulation in a depth-dependent manner. The median depth of the recording sites from the brain surface was 1412 μm, and the distance between most the superficial and deep sites at which neural activities were simultaneously recorded was 700 ± 194 μm (mean ± SD). To confirm whether modulatory effects were dependent on depth, we compared the PCI values of spontaneous activity between the recording sites located above and below the median depth from the brain surface. We performed this comparison for two points: 10 min before and after the drug administration and 40 min (all periods) before and after the drug administration. A Wilcoxon rank-sum test showed no significant difference in PCI between the two depths for both time periods in either the propranolol (10 min, *p* = 0.49, *Z* =  − 0.69; 40 min, *p* = 0.74, *Z* =  − 0.33) or saline conditions (10 min, *p* = 0.41, *Z* = 0.82; 40 min, *p* = 0.80, *Z* = 0.26). Moreover, a two-way ANOVA for repeated measures showed a significant decrease of the spontaneous activity after the propranolol administration, but no difference in the two depths and no interaction for both the 10-min (time, *F*_1, 61_ = 8.33, *p* < 0.01; depth, *F*_1, 61_ = 1.46, *p* = 0.23; interaction, *F*_1, 61_ = 0.006, *p* = 0.94) and 40-min (time, *F*_1, 61_ = 22.18, *p* < 0.0001; depth, *F*_1, 61_ = 1.81, *p* = 0.18; interaction, *F*_1, 61_ = 0.34, *p* = 0.56) observation periods. Thus, differential neuromodulation depending on the depth of recording sites was not observed.

**Fig. 4:**
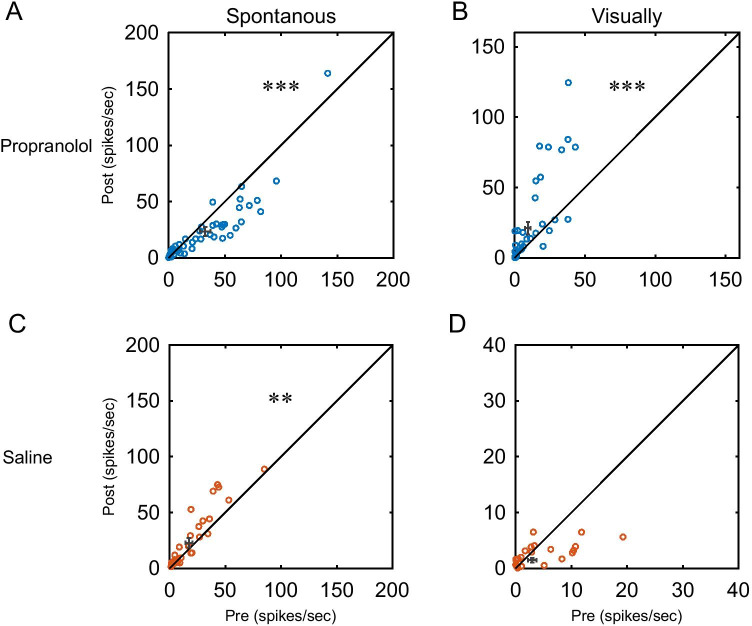
Population data of the drug effects on firing rates. **A**, **B** Each dot represents the firing rate of MUAs. Spontaneous activity (**A**) and visually evoked response (**B**) significantly decreased and increased, respectively, after propranolol administration. (*n* = 46; spontaneous activity, *Z* = 3.68, ***p < 0.001; visually evoked response, *Z* =  − 3.76, ***p < 0.001; Wilcoxon signed-rank test). **C**, **D** After saline administration, there was no significant change in the visually evoked response, but the spontaneous activity slightly but significantly increased (*n* = 35; spontaneous activity, *Z* =  − 3.08, **p < 0.01; visually evoked response, *Z* = 0.79, *p* = 0.43; Wilcoxon signed-rank test)

Figure 4 shows scatter plots of the firing rates, in which data for the post-drug administration are plotted against those for the pre-drug administration. At the population level, propranolol significantly decreased spontaneous activities and increased visual responses (*n* = 46; spontaneous activities, *Z* = 3.68, *p* < 0.001; visual responses, *Z* =  − 3.76, *p* < 0.001, Wilcoxon signed-rank test, Fig. 4A, B). On the other hand, saline administration caused no significant change in visual responses, but spontaneous activities slightly but significantly increased (*n* = 35; spontaneous activities, *Z* =  − 3.08, *p* < 0.01; visual responses, *Z* = 0.79, *p* = 0.43, Wilcoxon signed-rank test, Fig. 4C, D).

**Fig. 5:**
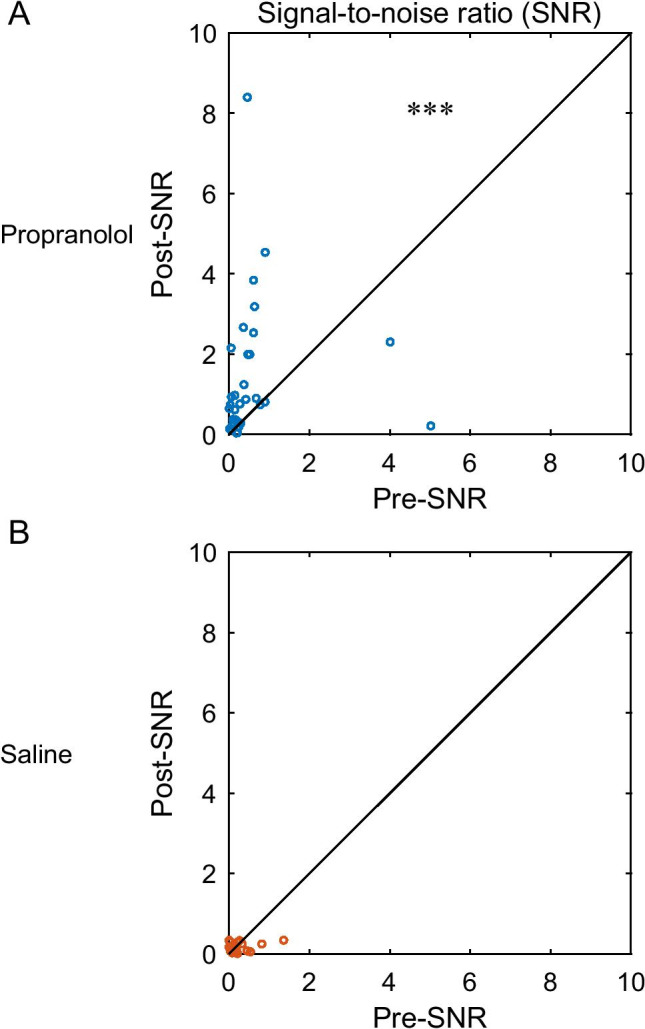
Population data of the drug effects on SNR in rat V1. **A** Propranolol significantly improved SNR at the population level (*n* = 46, *Z* =  − 3.32, ***p < 0.001, Wilcoxon signed-rank test). **B** Saline administration did not alter SNR (*n* = 35, *Z* = 1.05, *p* = 0.29, Wilcoxon signed-rank test)

The propranolol effects on spontaneous activities and visual responses led to the possibility that propranolol improved perceptual visual detectability by enhancing the SNR of neural activity in V1. To examine this point, we calculated the neuronal SNR. As expected, propranolol significantly increased the SNR (*n* = 46, *Z* =  − 3.32, *p* < 0.001, Wilcoxon signed-rank test, Fig. 5A), but saline control did not (*n* = 35, *Z* = 1.05, *p* = 0.29, Fig. 5B).

## Discussion

This study investigated the role of β-AR in visual detectability and the underlying neural mechanisms by combing drug administration and extracellular multi-point recordings in V1 of rats performing a VD task. We found that the topical administration of the β-AR antagonist propranolol to V1 (1) improved the perceptual visual detectability of low contrast stimuli, (2) decreased the spontaneous activity and increased the visual response in the MUA of V1, and (3) improved neuronal SNR. These findings suggest that NA modulates perceptual visual detectability by activating β-AR on V1 neurons. Thus, we demonstrated that a specific adrenergic receptor subtype mediates the NA modulation of neuronal SNR, regulating perceptual detectability.

### Noradrenaline and visual detectability

We demonstrated that the topical administration of propranolol onto the surface of V1 improved behavioral performance of the VD task. Recently, two studies investigated β-AR modulation on perceptual visual ability (Mizuyama et al. 2016; Treviño et al. 2019). Treviño et al. (2019) showed that the micro-infusion of isoproterenol, a β-AR agonist, in V1 decreased the visual discriminability of mice, indicating that the activation of β-AR in V1 worsens visual discriminability, which is consistent with our present study. On the other hand, our previous study (Mizuyama et al. 2016) in freely moving rats showed that propranolol decreased perceptual contrast sensitivity. What is the reason for the discrepancy? One explanation is the difference in the drug administration method. Propranolol topically administered onto the V1 surface diffuses several millimeters to sufficiently cover V1 but affects only a limited brain area (Goard and Dan 2009). On the other hand, Mizuyama and colleagues tested the effect of propranolol using an intraperitoneal administration, which spreads the drug throughout the whole brain and affects various visual areas including subcortical and cortical higher order areas. For example, Rogawski and Aghajanian (1980) showed that the iontophoretic administration of sotalol, a β-AR antagonist, on neurons in the lateral geniculate nucleus (LGN) of rats, suppresses NA-induced neuronal activities at low iontophoretic current, but increased NA-induced neuronal activities at higher iontophoretic currents. Therefore, the systemic administration of propranolol could suppress neuronal activities in LGN with low drug concentration. Further study on the effects of neuromodulators on several visual areas is necessary to understand the underlying network mechanism and functional role.

NA receptor agonists/antagonists are known to cause various kinds of modulatory effects on neuronal activity dose-dependently (Waterhouse et al. 1998; Devilbiss and Waterhouse 2000; Manella et al. 2017). Devilbiss and Waterhouse (2000) examined the effects of NA on neuronal responses in layer V neurons in the barrel cortex, a region of the primary somatosensory cortex, and showed that β-AR agonists suppress glutamate-induced neuronal excitation monotonically, implying that the inactivation of β-AR increases the cortical sensory response in a dose-dependent manner. However, the present study used only a single dose of the drug. Further, study testing multiple doses on visual detectability is required to ascertain how β-AR modulates visual function in a dose-dependent manner.

### Noradrenergic neuromodulation

In the electrophysiological analysis, the most prominent effect of propranolol was the augmentation of SNR. The SNR in sensory areas is the ratio between the response to the sensory input and baseline neural activity (Foote and Morrison 1987). Therefore, SNR can be improved by increasing the sensory response or by decreasing spontaneous activity. Based on signal detection theory (Tanner and Swets 1954), an improved SNR means less overlap between the probability distribution of the firing rate with the stimulus (signal distribution) and the probability distribution without the stimulus (noise distribution). There is a report showing the relationship between the augmentation of neuronal SNR and improved performance of a task requiring the detection of a stimulus with low presentation probability (Luo and Maunsell 2015). In that study, single-unit activity was recorded from V4 in monkeys performing an attention task, with attentional level controlled by manipulating the reward volume or target probability. The study found that attention modulated the SNR in V4 neurons, and the modulatory change corresponded well to that of the monkey’s task performance. Thus, the propranolol-induced SNR augmentation observed in the present study may explain the improved perceptual visual detectability.

The present study also found that SNR augmentation by propranolol is achieved by two modulatory effects: (1) decreased spontaneous activity and (2) increased visual response. Similar to our findings, Mueller et al. (1982) observed that NA suppressed spontaneous activities via β-AR in in vitro slice preparations of rat hippocampus. They reported that a β-AR agonist increased and a β-AR antagonist decreased the spontaneous activities of pyramidal neurons, indicating that intrinsic NA facilitates spontaneous activities via β-AR in the hippocampus. The β-AR-mediated facilitation of spontaneous activity has been reported to be caused by decreasing the amplitude and duration of afterhyperpolarization (AHP) (Rutecki 1995). Hence, the propranolol-induced suppression of spontaneous activity in the present study might be due to an increase of AHP.

Our results also demonstrated that visual responses in V1 are predominantly facilitated by propranolol. Devilbiss and Waterhouse (2000) found in in vitro tissue slice preparations of the rat somatosensory cortex that NA suppressed and facilitated glutamate-evoked discharges via β-AR and α-AR, respectively, which is consistent with our finding that the blockade of β-AR facilitated visual responses in V1. However, noradrenergic modulatory effects are known to vary depending on the cell type, as shown with somatostatin-immunoreactive and cholecystokinin-immunoreactive cells (Kawaguchi and Shindou 1998). Therefore, the diversity of the NA modulatory effects might be explained by differences in cell type. Further study is required to clarify the whole neural mechanism that leads to perceptual change.

### Functional role of noradrenaline

The modulatory effects of NA on sensory information processing (Devilbiss and Waterhouse 2000; Devilbiss et al. 2006; Manella et al. 2017) and perception (Rajkowski et al. 1994; Usher et al. 1999) are unique, showing an inverted U-shaped function against NA concentration or LC activity. For example, moderate levels of NA concentration or LC activity caused the facilitation of sensory responses or correlated with improved behavioral task performance, but levels too low or too high inhibited sensory responses and lowered task performance (Aston-Jones and Cohen 2005; McBurney-Lin et al. 2019). The complexity of the modulatory effects has been ascribed to the diversity of AR subtypes, which have distinct properties. There are three AR subtypes (*α*_1_, *α*_2_, and *β*) expressed in the brain, and their affinities to NA differ (*α*_1_, ≈300 nM; *α*_2_, ≈50 nM; and *β*, ≈ 0.7–0.8 µM) (Ramos and Arnsten 2007; Salgado et al. 2016). Given the different affinities of these AR subtypes, the concentration of NA should be a determinant of the NA modulation. Indeed, several studies observed that the facilitatory and inhibitory NA modulatory effects are mimicked by selective agonists of α_1_- and β-AR, respectively (Devilbiss and Waterhouse 2000). Regarding vision, some studies observed different AR-subtype effects on neuronal activities in V1 (Sato et al. 1989) and perception (Mizuyama et al. 2016; Treviño et al. 2019). To elucidate the AR modulation of neuronal activity and behavioral performance in the VD task, further study focused on α_1_-AR and α_2_-AR is needed.

Our finding indicates that intrinsic NA suppresses perceptual and neuronal visual detectability via β-AR in V1. Taken together with a previous finding showing that β-AR has a low affinity for NA (0.7–0.8 µM; Ramos and Arnsten 2007), a high concentration of NA is needed to activate β-AR. In other words, β-AR might exert a modulatory effect at the high activity of LC. What is the physiological significance of β-AR suppressive modulation on visual function? In general, the more animals are stressed, the more LC is tonically activated (Tanaka et al. 1983; Berridge and Waterhouse 2003; Valentino and Van Bockstaele 2008; Chamberlain and Robbins 2013). Kane et al. (2017) reported that artificially induced tonic LC activity in rats causes disengagement from current behavior and pursuit alternatives in a patch-foraging task. They concluded that NA released by tonic LC activity facilitated the task disengagement by increasing decision noise. Our finding that NA reduces SNR by increasing spontaneous activity suggests that a high NA concentration disrupts the sensory response via β-AR, leading to the disengagement of any ongoing goal-directed behavior. Thus, the NA-induced control of a sensory response may promote a behavioral shift that can be adaptive to hyperarousal or stressful circumstances.

### Technical limitations

In this study, we tested drug effects by topical administration on the V1 surface. Thus, we cannot rule out the possibility that the drug diffused to and affected other brain areas. However, a previous rat study reported that the topical administration of AR antagonists including propranolol onto the cortical surface of the parietal lobe did not affect regional cerebral blood flow, neural activity 2–3 mm away from the drug administration site in the same brain hemisphere, or systemic arterial blood pressure, suggesting topical administration is a suitable local drug administration method (Richter et al. 2005). The 2–3 mm around our craniotomy (3.7 mm posterior to the bregma, 7.3 mm lateral, diameter: 1.5–2 mm) is mostly occupied by V1 (Paxinos and Watson 2007), with some higher visual areas. Thus, these evidences suggest that the effect of propranolol was limited in V1 or, at most, higher visual areas. The representative peripheral effects of propranolol are a decrease in sympathetic activity, causing a decrease in heart rate or blood pressure, dry eyes, and drowsiness (Singer et al. 1984; Frcka and Lader 1988; Léaute-Labrèze et al. 2016), resulting in increased reaction time in the task. Neither dry eyes nor an increase in reaction time compared to the saline condition were observed by the propranolol administration, suggesting that any peripheral effects were negligible on the task performance.

### Conclusion

In conclusion, the present study found that the blockade of β-AR improves the SNR in awake rat V1, resulting in improved visual detectability. Although the local administration of a β-AR antagonist to V1 has been previously shown to produce neural modulation in anesthetized animals, the present study is the first to show the direct relationship between β-adrenergic neural modulation and changes in the perceptual performance of awake animals. This result suggests that visual information processing in V1 is dynamically modulated in a NA-dependent manner to achieve adaptive behavior.

## Abbreviations

LC: Locus coeruleus
NA: Noradrenaline
AR: Adrenergic receptor
V1: Primary visual cortex
SNR: Signal-to-noise ratio
MUA: Multi-unit activity
